# Factors determining chance of type 2 diabetes remission after Roux-en-Y gastric bypass surgery: a nationwide cohort study in 8057 Swedish patients

**DOI:** 10.1136/bmjdrc-2020-002033

**Published:** 2021-05-14

**Authors:** Erik Stenberg, Torsten Olbers, Yang Cao, Magnus Sundbom, Anders Jans, Johan Ottosson, Erik Naslund, Ingmar Näslund

**Affiliations:** 1 Faculty of Medicine and Health, Örebro Universitet, Örebro, Sweden; 2 Department of Biomedical and Clinical Sciences and Wallenberg Center for Molecular Medicine, Linköping University, Linkoping, Sweden; 3 Department of Gastrosurgical Research, Sahlgrenska University Hospital, Goteborg, Sweden; 4 Clinical Epidemiology and Biostatistics, School of Medical Sciences, Örebro University, Orebro, Sweden; 5 Department of Surgical Sciences, Uppsala University, Uppsala, Sweden; 6 Division of Surgery, Department of Clinical Sciences, Karolinska Institutet, Stockholm, Sweden

**Keywords:** diabetes mellitus, type 2, bariatric surgery

## Abstract

**Introduction:**

Bariatric and metabolic surgery is an effective treatment option for type 2 diabetes (T2D). Increased knowledge regarding factors associated with diabetes remission is essential in individual decision making and could guide postoperative care. Therefore, we aimed to explore factors known to affect the chance of achieving diabetes remission after bariatric and metabolic surgery and to further investigate the impact of socioeconomic factors.

**Research design and methods:**

In this nationwide study, we assessed all patients with T2D who underwent Roux-en-Y gastric bypass (RYGB) surgery between 2007 and 2015 in the Scandinavian Obesity Surgery Registry. Remission was defined as absence of antidiabetic medication for T2D 2 years after surgery. Multivariable logistic regression was used to evaluate factors associated with diabetes remission, with missing data handled by multiple imputations.

**Results:**

A total of 8057 patients were included. Mean age±SD was 47.4±10.1 years, mean body mass index 42.2±5.7 kg/m^2^, mean hemoglobin A1c 59.0±17.33, and 61.7% (n=4970) were women. Two years after surgery, 6211 (77.1%) patients achieved T2D remission. Preoperative insulin treatment (OR 0.26, 95% CI 0.22 to 0.30), first-generation immigrant (OR 0.66, 95% CI 0.57 to 0.77), duration of T2D (OR 0.89, 95% CI 0.88 to 0.90), dyslipidemia (OR 0.71, 95% CI 0.62 to 0.81), age (OR 0.97, 95% CI 0.96 to 0.97), and high glycosylated hemoglobin A1c (HbA1c) (OR 0.99, 95% CI 0.98 to 0.99) were all associated with lower T2D remission rate. In contrast, residence in a medium-sized (OR 1.39, 95% CI 1.20 to 1.61) or small (OR 1.46, 95% CI 1.25 to 1.71) town and percentage of total weight loss (OR 1.04, 95% CI 1.03 to 1.04) were associated with higher remission rates.

**Conclusion:**

Among patients with T2D undergoing RYGB surgery, increasing age, HbA1c, and diabetes duration decreased the chance of reaching diabetes remission without cut-offs, while postoperative weight loss demonstrated a positive linear association. In addition, being a first-generation immigrant and living in a large city were socioeconomic factors having a negative association.

**Significance of this study**

**What is already known about this subject?**

- While bariatric and metabolic surgery offers a high chance of diabetes remission, previous studies have primarily evaluated factors associated with remission when separated into prespecified categories.
- The contribution of socioeconomic factors is incompletely investigated.

**What are the new findings?**

- Factors with known association with diabetes remission had a linear inverse relationship with type 2 diabetes (T2D) remission without clear cut-off.
- Socioeconomic factors were relevant for diabetes remission.

**How might these results change the focus of research or clinical practice?**

- The results of the study add to the body of literature supporting bariatric and metabolic surgery for patients with severe obesity and T2D.

## Introduction

Type 2 diabetes (T2D) is a major threat to public health, with an estimated global prevalence of 9.8% in men and 9.2% in women.^1^ A high but stable incidence of T2D has been reported in many countries in recent years,^2^ and obesity remains one of the strongest risk factors for T2D.^3^ In addition, diabetes has been reported to be the second major body mass index (BMI)-related cause of death, accounting for approximately 0.9 million deaths annually worldwide.^4^

Currently, bariatric and metabolic surgery is associated with a low risk of serious complications and side effects, and well-documented long-term weight loss and improvements in many relevant obesity-related comorbidities.^5–7^ The benefits of surgery appear to be particularly high for patients with T2D, where diabetes remission is common.^5 8 9^ Although diabetes relapse occurs in the long run, surgical treatment reduces the risk of diabetes complications and reduces the cost of healthcare.^5 8–11^

Despite the excellent results of bariatric and metabolic surgery, only a small proportion of eligible patients with T2D are evaluated for this treatment.^12^ Furthermore, there is often limited access to surgery based on perceived low rates of T2D remission in older patients and advanced disease. Improved knowledge on factors associated with the chance of remission would enable us to properly inform patients who may benefit from bariatric and metabolic surgery.

Duration of diabetes, glycemic control, use of insulin, older age, and degree of postoperative weight loss have all been reported as factors influencing the chance of diabetes remission.^8 13–15^ However, most of these factors have been evaluated with cohorts divided into categories, often leading to some cut-off values for bariatric and metabolic surgery indications. One example is the recommendation of a 10-year duration of diabetes limit suggested by the British National Institute for Health and Care Excellence.^16^ Recent data have suggested a more linear relationship between diabetes remission and diabetes duration in a cohort with a duration from 0 to 26 years, bringing into question recommendations based on specific thresholds.^13^

Socioeconomic factors generally viewed as indicators of a low socioeconomic status are known to increase the incidence of T2D, in particular in high-income countries.^17^ Recent studies have also reported an association between several socioeconomic factors and the risk for complications, as well as the effect on weight loss and improvements in health-related quality of life after bariatric surgery.^18–20^ However, the effect of socioeconomic factors on remission rates of T2D after bariatric surgery remains unknown.

This study aimed to explore further preoperative factors known to affect the chance of achieving diabetes remission after bariatric and metabolic surgery and to investigate the impact of socioeconomic factors.

## Methods

The Scandinavian Obesity Surgery Registry (SOReg) is a national research and quality database covering virtually all bariatric and metabolic surgical procedures in Sweden.^21^ By using personal identification numbers (unique to all Swedish citizens), the SOReg database was linked to the Swedish National Patient Register, the Swedish National Death Register, the Swedish Prescribed Drug Register, and Statistics Sweden. These registers cover inpatient and outpatient hospital visits, mortality, drugs prescribed, and individual socioeconomic data. All patients who underwent a primary bariatric and metabolic surgical procedure (Roux-en Y gastric bypass or sleeve gastrectomy) between 2007 and 2015 and registered in the SOReg were considered for inclusion. Only patients diagnosed with T2D, as defined by the American Diabetes Association (ie, fasting plasma glucose ≥126 mg/L (7.0 mmol/L), glycosylated hemoglobin A1c (HbA1c) ≥48 mmol/mol (6.5%), or medical treatment for diabetes), operated with a primary Roux-en-Y gastric bypass (RYGB) procedure were included in the study.^22^

The SOReg reports metabolic obesity-related diseases, defined as medical treatment for a specific metabolic obesity-related disease (diabetes, hypertension, dyslipidemia, dyspepsia/gastroesophageal reflux disease, and depression), and nocturnal continuous positive airway pressure treatment for sleep apnea. Since cardiovascular comorbidity is not a mandatory variable in the SOReg, data on this comorbidity were based on combined data from the Swedish National Patient Register and the SOReg, defined as a history of ischemic heart disease, angina pectoris, cardiomyopathy, cardiac failure, or cardiac arrhythmia. Diabetes duration was based on a combination of data from the SOReg, the Swedish National Patient Register, and the Swedish Prescribed Drug Register, while HbA1c values were reported at predefined outpatient visits. SOReg data are prospectively collected at baseline about 1 month before surgery, and on day 30 and at 1, 2, 5, and 10 years after surgery. Most registrations are based on information from consultations at the outpatient clinic. When this is not possible, follow-ups are based on telephone, e-meetings, or written response, with blood samples and weights registered from primary care or a different hospital outpatient clinic.

Educational level was divided into four categories based on the highest completed education at the time of surgery: primary education (≤9 years of schooling), secondary education (completed 10–12 years of schooling), higher education ≤3 years (completed college or university degree with ≤3 years of education), and higher education >3 years. Disposable income (total taxable income minus taxes and other negative transfers) was divided into percentiles (lowest 20th, 20th to median, median to 80th, and highest 80th) based on the disposable income of all adults in Sweden during the year of surgery. Place of residence was divided into three categories, following definitions used by the Swedish Association of Local Authorities and Regions: large city (>200 000 inhabitants) or municipality near a large city; medium-sized town (≥50 000 inhabitants) or municipality near a medium-sized town; and smaller town or urban area (<50 000 inhabitants) or rural municipality. Heritage was divided into three categories based on the country of birth and parents’ place of birth, as described previously.^18^

Postoperative weight loss at 2 years after surgery was presented as change in BMI (BMI loss=initial BMI–postoperative BMI), percentage of total weight loss (TWL=100×weight loss/preoperative weight), and percentage of excess BMI loss (EBMIL=100×[initial BMI–postoperative BMI]/[initial BMI–25]). All baseline values were evaluated before preoperative weight reduction.

Postoperative complications were classified according to the Clavien-Dindo classification of postoperative complications, with any complication defined as Clavien-Dindo ≥1 (ie, any deviation from a normal postoperative course).^23^ Specification of the severity of complications was introduced in the SOReg in 2010, and serious postoperative complications (defined as Clavien-Dindo ≥3b, ie, need for intervention requiring general anesthesia or intensive care due to single-organ or multiorgan failure, or mortality) were analyzed for patients operated after 2010.

### Outcomes

The main outcome was remission of diabetes 2 years after surgery. Remission was defined as being without diabetes medication within a time frame of ±6 months, ie, 18–30 months postoperatively. Secondary outcomes were complete or partial remission of diabetes. Complete remission was defined as an HbA1c value ≤42 mmol/mol without medical treatment, and partial remission was defined as an HbA1c value ≤48 mmol/mol without medical treatment.^24^

### Statistics

Categorical data are presented as numbers (n) and percentages (%), and continuous variables as mean±SD. Univariable, conditional, logistic regression and multivariable, conditional, logistic regression (including age, sex, BMI, HbA1c, and insulin treatment at baseline, obesity-related disease, and TWL 2 years after surgery) were used to assess risks reported as OR with 95% CI as measures of association. Socioeconomic variables were analyzed using univariable, logistic regression and multivariable, logistic regression with adjustment for patient-specific variables associated with the chance of achieving diabetes remission in the previous multivariable analysis (defined as p<0.05 after correction for multiple calculations). The Bonferroni-Holm method was used to correct for multiple calculations when appropriate.^25^ Missing data were handled by multiple imputations (presented in the manuscript), while analyses based on listwise deletion are presented in online supplemental tables 1–3; however, only minor differences were observed.

10.1136/bmjdrc-2020-002033.supp1 Supplementary data

SPSS Statistics V.25 was used for all statistical analyses.

## Results

During the inclusion period from 2007 to 2015, 8112 patients with T2D who underwent RYGB procedures were identified in the SOReg. After excluding 55 individuals who died within 2 years after surgery, 8057 patients remained in the study. At 2-year follow-up, weight and BMI were registered for 4997 patients (62.0%). A registration of HbA1c 12–24 months after surgery was available for 6438 patients (79.4%). The baseline characteristics of the study participants are presented in table 1.

**Table 1:** Baseline characteristics of the study group

	Missing values (%)	Entire group	Remission	Non-remission	P value
		n (%), mean±SD	n (%), mean±SD	n (%), mean±SD	
Age, years	0	47.4±10.08	46.6±10.2	51.7±8.7	<0.001
Sex	0				
Female		4970 (61.7)	3891 (62.6)	1079 (58.5)	Ref
Male		3087 (38.3)	2320 (37.4)	767 (41.5)	0.001
Weight, kg	0	122.7±21.74	123.6±22.01	119.7±20.50	<0.001
Waist circumference, cm	1370 (17.0)	129.5±13.21	129.7±13.3	128.7±12.77	0.011
BMI, kg/m^2^	0	42.2±5.74	42.5±5.8	41.2±5.4	<0.001
Diabetes duration	0	3.7±4.59	2.7±3.6	7.2±5.8	<0.001
Glycosylated hemoglobin A1c, mmol/mol	1068 (13.3)	59.0±17.33	56.7±16.5	67.3±17.5	<0.001
Median number of antidiabetic drugs	0	1 (1–2)*	1 (0–2)*	2 (1–2)*	<0.001
Insulin treatment	0	2308 (28.6)	669 (11.6)	1177 (51.0)	<0.001
Obesity-related disease	0				
Dyslipidemia		2527 (31.4)	1663 (26.8)	864 (46.8)	<0.001
Dyspepsia/GERD		1031 (12.8)	761 (12.3)	270 (14.6)	0.007
Depression		1297 (16.1)	986 (15.9)	311 (16.8)	0.318
Sleep apnea		1529 (19.0)	1146 (18.5)	383 (20.7)	0.027
Hypertension		4546 (56.4)	3259 (52.5)	1287 (69.7)	<0.001
Cardiovascular comorbidity		917 (11.4)	612 (9.9)	305 (16.5)	<0.001
Education	55 (0.7)				
Primary education ≤9 years		1606 (19.9)	1214 (19.7)	392 (21.4)	0.219
Secondary education		4762 (59.1)	3671 (59.5)	1091 (59.5)	Ref
Higher education ≤3 years		838 (10.4)	659 (10.7)	179 (9.8)	0.323
Higher education >3 years		796 (9.9)	623 (10.1)	173 (9.4)	0.463
Disposable income	86 (1.1)				
<20th percentile		2239 (27.8)	1715 (27.9)	524 (28.8)	0.285
20th–50th percentile		2520 (31.3)	1963 (31.9)	557 (30.6)	Ref
50th–80th percentile		2254 (28.0)	1757 (28.6)	497 (27.3)	0.965
>80th percentile		958 (11.9)	714 (11.6)	244 (13.4)	0.035
Residence	31 (0.4)				
Large city or municipality		2734 (33.9)	2047 (33.1)	687 (37.2)	Ref
Medium-sized town or municipality		3061 (38.0)	2390 (38.7)	671 (36.4)	0.004
Small town, urban area, rural municipality		2231 (27.7)	1744 (28.2)	487 (26.4)	0.007
Heritage	18 (0.2)				
Swedish-born, Swedish descendant		6072 (75.4)	4727 (76.3)	1345 (72.9)	Ref
Swedish-born, non-Swedish descendant		338 (4.2)	267 (4.3)	71 (3.8)	0.621
Born outside Sweden		1629 (20.2)	1200 (19.4)	429 (23.3)	<0.001

*Median (interquartile range).

BMI, body mass index; GERD, gastroesophageal reflux disease; Ref, reference.

### Surgical data

Most surgical procedures were laparoscopic (n=7745, 95.5%), 257 were primary open procedures (3.2%), and 110 were conversions to open surgery (1.4%). Postoperative complications occurred in 821 operations (10.2%). For patients operated after 2010, serious postoperative complications, requiring intervention under general anesthesia or intensive care, occurred in 223 operations (2.7%). The mean BMI loss 2 years after surgery was 12.0±4.5 kg/m^2^, with TWL of 28.4%±8.9% and EBMIL of 73.3%±24.4%.

### Effects on diabetes

**Figure 1:**
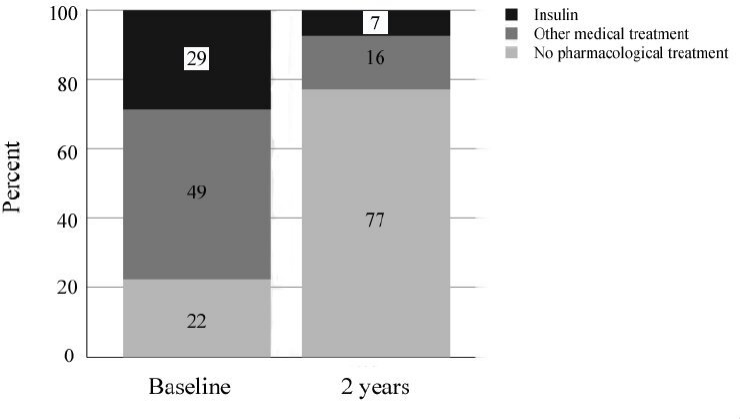
Use of antidiabetic drugs before and at 2 years after surgery. Percentage of all patients (N=8057) at each point.

At baseline, 2313 patients (28.7%) were treated with insulin, while 3940 were given medical treatment not including insulin (48.9%), and 1804 received non-pharmacological treatment only (22.4%). Two years after surgery, 595 patients (7.4%) received insulin treatment, 1251 received medical treatment not including insulin (15.5%), and 6211 (77.1%) received no treatment (figure 1).

Two years after surgery, 4004 patients (62.2%) achieved complete diabetes remission, 680 patients (10.6%) achieved partial remission, and 687 patients (10.7%) had an HbA1c level <48 mmol/mol with medical treatment, while 1067 patients (16.6%) still had an HbA1c ≥48 mmol/mol on medical treatment.

Two years after surgery, 1129 patients (48.8%) who received insulin at baseline did not receive pharmacological treatment for diabetes, compared with 5082 patients (88.5%) who were not receiving insulin at baseline, while 577 patients (24.9%) initially receiving insulin still required insulin treatment at 2-year follow-up.

**Figure 2:**
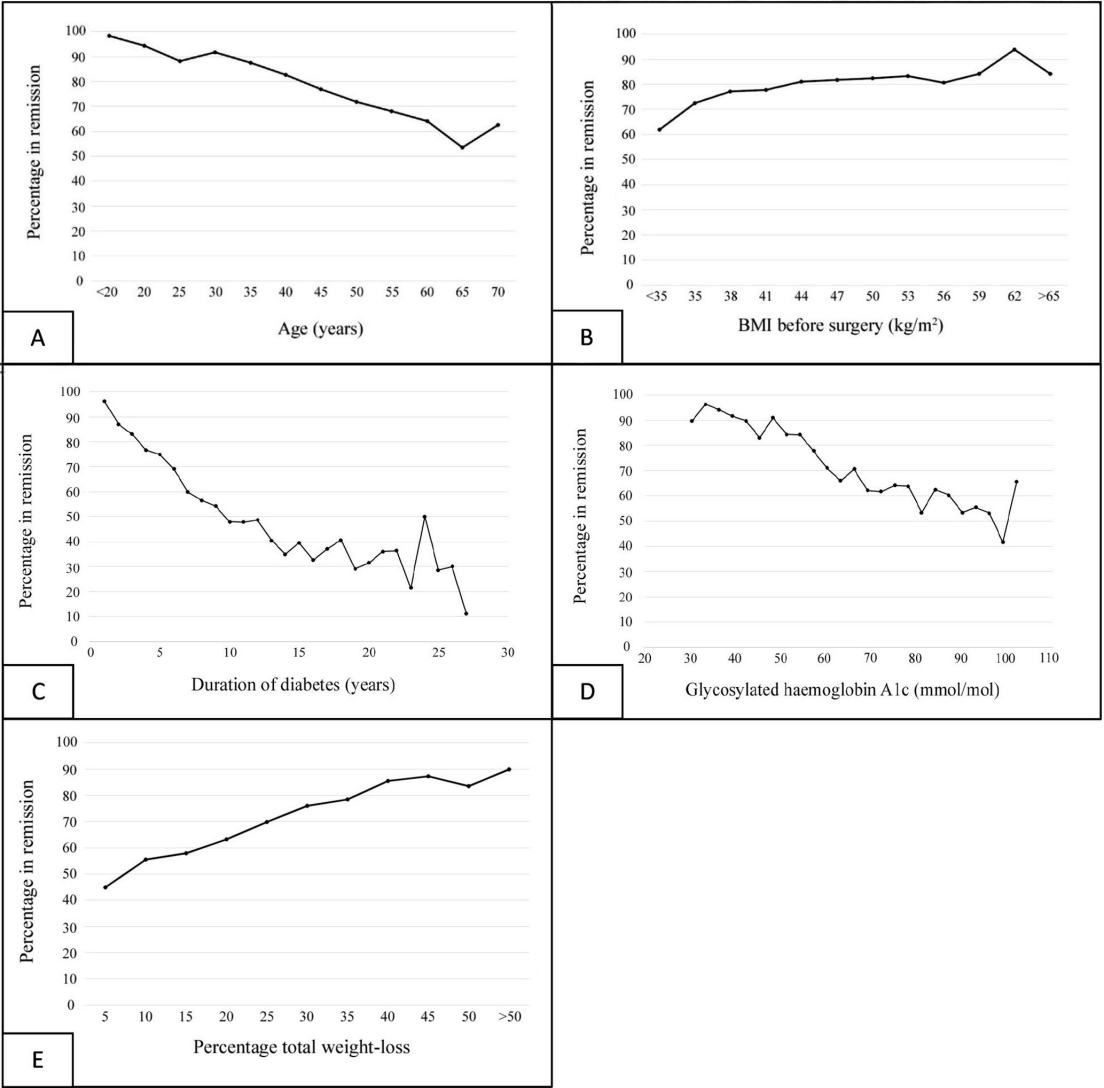
Association between continuous variables and cessation of treatment for T2D. Shown are age (A; Spearman coefficient −0.21, p<0.0001), BMI before surgery (B; Spearman coefficient 0.11, p<0.001), duration of T2D (C; Spearman coefficient −0.43, p<0.001), glycosylated hemoglobin A1c (D; Spearman coefficient −0.28, p<0.001), and postoperative total weight loss (E; Spearman coefficient 0.19, p<0.001). BMI, body mass index; T2D, type 2 diabetes.

Age, BMI at baseline, duration of diabetes, preoperative HbA1c level, and postoperative weight loss were all continuous variables affecting the chance of diabetes remission in the univariable analyses (table 2). The association assumed a linear correlation for these variables (figure 2). No statistical association was observed for BMI in the multivariable analyses (table 2). The categorical variables insulin treatment at baseline and dyslipidemia were also negatively associated with diabetes remission (table 2).

**Table 2:** Chance of reaching diabetes remission 2 years after surgery

	Unadjusted OR (95% CI)	Adjusted OR (95% CI)*	Adjusted p value*
Age	0.95 (0.94 to 0.95)	0.97 (0.96 to 0.97)	<0.001
Sex			
Female	Reference	Reference	Reference
Male	0.84 (0.76 to 0.93)	1.10 (0.96 to 1.26)	0.158
BMI	1.05 (1.04 to 1.06)	1.00 (0.99 to 1.01)	0.848
Diabetes duration	0.80 (0.79 to 0.81)	0.89 (0.88 to 0.90)	<0.001
Glycosylated hemoglobin A1c	0.97 (0.96 to 0.97)	0.99 (0.98 to 0.99)	<0.001
Insulin treatment at baseline	0.12 (0.11 to 0.14)	0.26 (0.22 to 0.30)	<0.001
Percentage of total weight loss	1.04 (1.03 to 1.05)	1.04 (1.03 to 1.04)	<0.001
Obesity-related disease			
Dyslipidemia	0.42 (0.37 to 0.46)	0.71 (0.62 to 0.81)	<0.001
Dyspepsia/GERD	0.82 (0.70 to 0.95)	0.99 (0.83 to 1.19)	0.927
Depression	0.93 (0.81 to 1.07)	0.91 (0.76 to 1.07)	0.248
Sleep apnea	0.86 (0.76 to 0.98)	1.02 (0.87 to 1.20)	0.785
Hypertension	0.48 (0.43 to 0.54)	0.88 (0.76 to 1.01)	0.078
Cardiovascular comorbidity	0.55 (0.48 to 0.64)	0.98 (0.82 to 1.18)	0.864

*Multivariable logistic regression including all factors listed in the table, with multiple imputation for missing values.

BMI, body mass index; GERD, gastroesophageal reflux disease.

### Socioeconomic factors

The highest percentiles of disposable income were associated with a lower chance of reaching diabetes remission, but the association did not remain significant after correction for multiple calculations. Residence in a large city and being a first-generation immigrant were factors associated with a lower chance of achieving diabetes remission (table 3).

**Table 3:** Chance of reaching diabetes remission depending on socioeconomic status

	Unadjusted OR (95% CI)	Adjusted OR (95% CI)†	Adjusted p value†
Education			
Primary education ≤9 years	0.92 (0.81 to 1.05)	0.96 (0.82 to 1.13)	1.000*
Secondary education	Reference	Reference	Reference
Higher education ≤3 years	1.09 (0.91 to 1.31)	1.06 (0.86 to 1.31)	1.000*
Higher education >3 years	1.07 (0.89 to 1.28)	1.00 (0.80 to 1.24)	0.891*
Disposable income			
<20th percentile	0.93 (0.81 to 1.06)	0.86 (0.73 to 1.01)	0.116*
20th–50th percentile	Reference	Reference	Reference
50th–80th percentile	1.00 (0.87 to 1.15)	0.98 (0.84 to 1.16)	0.796*
>80th percentile	0.83 (0.70 to 0.99)	0.80 (0.65 to 0.99)	0.069*
Residence			
Large city and municipality (>200 000 inhabitants)	Reference	Reference	Reference
Medium-sized town and municipality (50 000–200 000 inhabitants)	1.20 (1.06 to 1.35)	1.39 (1.20 to 1.61)	<0.001*
Small town, urban area, rural municipality (<50 000 inhabitants)	1.20 (1.05 to 1.37)	1.46 (1.25 to 1.71)	<0.001*
Heritage			
Swedish-born, Swedish descendant	Reference	Reference	
Swedish-born, non-Swedish descendant	1.10 (0.84 to 1.43)	0.82 (0.60 to 1.13)	0.235*
Born outside Sweden	0.80 (0.70 to 0.90)	0.66 (0.57 to 0.77)	<0.001*

*P values after correction for multiple comparisons with the Bonferroni-Holm method.

†Adjusted for age, sex, BMI, insulin treatment at baseline, dyslipidemia, duration of diabetes, HbA1c at baseline, and %TWL at 2-year follow-up, with multiple imputation for missing values.

BMI, body mass index; HbA1c, glycosylated hemoglobin A1c; %TWL, percentage of total weight loss.

## Discussion

At 2-year follow-up after bariatric and metabolic surgery, we could verify several factors associated with a lower chance of T2D remission rate: duration of diabetes, HbA1c, and older age. However, being a first-generation immigrant and a resident in a large city was associated with lower remission rates. Large postoperative weight loss was associated with a better chance of diabetes remission.

High HbA1c at baseline, insulin treatment, longer duration of T2D, and metabolic comorbidities such as dyslipidemia all represented markers of poor control of metabolic diseases and had previously been described as factors associated with a lower chance of diabetes remission.^13^ A linear, inverse relationship between duration of diabetes and the chance of achieving remission has previously been suggested and was confirmed in this study.^13^ Moreover, we could demonstrate a similar linear inverse relationship regarding preoperative HbA1c and age, whereas we found a positive linear relationship between postoperative total weight loss and remission.

Age was previously reported to be negatively associated with the chance of achieving diabetes remission.^13^ Older patients were more likely to have obesity-related disease, less physically active, and achieved less weight loss after surgery than younger patients. An inverse linear correlation was observed between age and chance of T2D remission in this present study, although we did not find any clear cut-off. Even the group of patients aged >60 years showed a high remission rate (in our study as well as in the previous literature), suggesting that age should not limit access to bariatric and metabolic surgery.^26^

A longer duration of T2D, and disease severity, is usually associated with impaired beta-cell function and insulin resistance. Although the function of beta-cells may improve after bariatric surgery, damage can be irreversible.^27^ Even if some patients with severe T2D do not achieve complete remission, they may benefit from a reduction in insulin resistance and partial beta-cell recovery. Indeed, about 75% of patients with insulin treatment at baseline had discontinued insulin use 2 years after surgery.

The mechanisms of action mediating the positive effects of bariatric and metabolic surgery are only partially understood; however, weight loss is an obvious contributing factor.^27^ A high BMI at baseline is associated with high insulin resistance, and significant improvements in weight-dependent disease after surgery have previously been reported. In addition, the higher chance of diabetes remission in patients with higher BMI, seen in the present univariable analysis, disappeared in the multivariable analyses (including supplementary analysis with stratified BMI as presented in online supplemental tables 1–3), indicating that weight loss alone may be a more important factor than BMI.

In addition to previously known factors, this study suggested that socioeconomic factors are important determinants of the chance of diabetes remission after bariatric and metabolic surgery. First-generation immigrants had a lower chance of diabetes remission, as had patients residing in a larger city. An association between both of these factors and the risk of postoperative complications has been previously reported.^18^ Previous studies have reported lower weight loss and improvement in health-related quality of life, as well as higher loss to follow-up after bariatric surgery for patients living in larger cities.^19 20 28^ While part of these findings may be explained by the chronic stress and higher cortisol levels of urban life, as well as the higher availability of energy-dense food, the cause of these negative associations remains largely unknown.^29^ However, low socioeconomic status and income are associated with poor health literacy, factors known to influence postoperative recovery and treatment compliance.^30 31^ Furthermore, access to healthcare may differ depending on socioeconomic status. We recently demonstrated that lower socioeconomic status precludes patients with T2D in general from having RYGB in Sweden.^32^ Further research is needed to understand these inequalities in health outcomes, but an enhanced perioperative and postoperative support for certain groups may improve long-term outcomes.

Diabetes currently causes the highest financial load on the healthcare system, with >50% of costs being on medications.^33^ Although the initial costs of bariatric and metabolic surgeries are substantial, the subsequent overall reduction in diabetes medication and complications represents reduced costs over time, even with less effective surgical methods than those used today.^34^

Although the results of the present study suggest that younger patients with a short duration of diabetes and better glucose control before insulin treatment have the best chance of achieving diabetes remission, surgical treatment should not be discouraged for older patients with longer diabetes duration and/or poor diabetes control. These results contradict the widespread opinion that bariatric and metabolic surgery should be considered a ‘last resort’ when all other treatment options have failed. Metabolic surgery for patients with T2D should rather be considered early in the course of the disease. This has been concluded in the Swedish Obese Subjects Study and further emphasized in the Second Diabetes Surgery Summit recommendations.^5 35^ However, even patients not reaching full remission may benefit substantially from surgery, that is, reduction in dose and number of drugs (particularly insulin) and reduction in other comorbidities such as osteoarthritis and obstructive sleep apnea. Therefore, limited access to bariatric and metabolic surgeries based on age, BMI, and duration and severity of diabetes should be used with caution.

### Limitations

The combination of several high-quality registers in this study provided almost complete follow-up with the highest quality data. However, the main limitation was our definition of remission based on medical treatment alone with no confirmation using laboratory values for the main outcome, potentially overestimating treatment effects. Patients in diabetes remission may remain on metformin because of their possible protection against cardiovascular events and relapse of disease, thus underestimating the correct remission rate.^36 37^ In addition, follow-up for the main outcome was limited to 2 years. This short-term follow-up was decided because the majority of remission would occur during this period. A longer follow-up period would allow the inclusion of relapse of disease. C peptide has previously been reported as a factor associated with a reduced chance of achieving diabetes remission.^38^ Since C peptide is not generally registered in the SOReg, it could not be included in this study. Finally, due to the low historical number of sleeve gastrectomies in Sweden, only RYGB was evaluated. Although this has the strength of avoiding differences in associations and efficacy between surgical methods, it limits generalizability to the surgical method evaluated.

## Conclusion

Among patients with T2D undergoing gastric bypass surgery, the overall remission rate, defined as being off medication, was 77%. Factors known to be associated with a lower remission rate, such as duration of diabetes, older age, and severity of metabolic impairment, had a linear inverse relationship with remission of T2D. Some socioeconomic factors were also associated with a lower chance of achieving T2D remission in this study: first-generation immigrants and living in a large city. Large postoperative weight loss was associated with a better chance of remission.

## Data Availability

Data may be obtained from a third party and are not publicly available. Data cannot be shared publicly due to patient confidentiality under current Swedish legislation. Data are available from the Scandinavian Obesity Surgery Registry (contact via www.ucr.uu.se/soreg/) for researchers who meet the criteria for access to confidential data.
