# Supplementary material for: Factors determining chance of type 2 diabetes remission after Roux-en-Y gastric bypass surgery: a nationwide cohort study in 8057 Swedish patients
Source: BMJ Open Diabetes Res Care. 2021 May 14;9(1):e002033. doi: 10.1136/bmjdrc-2020-002033 (PMC8127970; doi:10.1136/bmjdrc-2020-002033)
Supplement: Supplementary data [file bmjdrc-2020-002033supp001.pdf]

Supplementary Table 1

Supplementary Table 1. Chance of reaching diabetes remission 2 years after surgery

	Unadjusted OR (95% CI)	Adjusted OR (95%CI)[1]	Adjusted-P[1]
Age	0.95 (0.94-0.95)	0.96 (0.95-0.97)	<0.001
Sex			
Female	Reference	Reference	Reference
Male	0.84 (0.76-0.93)	1.33 (1.11-1.60)	0.003
BMI	1.05 (1.04-1.06)	0.99 (0.97-1.00)	0.093
Diabetes duration	0.80 (0.79-0.81)	0.89 (0.87-0.91)	<0.001
Glycosylated Haemoglobin A1c	0.97 (0.96-0.97)	0.99 (0.98-0.99)	<0.001
Insulin treatment at baseline	0.12 (0.11-0.14)	0.22 (0.18-0.26)	<0.001
Percentage Total weight-loss	1.05 (1.04-1.06)	1.06 (1.05-1.07)	<0.001
Obesity-related disease			
Dyslipidaemia	0.42 (0.37-0.46)	0.65 (0.54-0.78)	<0.001
Dyspepsia/GERD	0.82 (0.70-0.95)	0.90 (0.69-1.17)	0.432
Depression	0.93 (0.81-1.07)	1.13 (0.88-1.46)	0.332
Sleep apnoea	0.87 (0.76-0.98)	0.97 (0.78-1.22)	0.818
Hypertension	0.48 (0.43-0.54)	0.93 (0.76-1.13)	0.465
Cardiovascular comorbidity	0.55 (0.46-0.66)	1.08 (0.83-1.42)	0.556

1 - Multivariable logistic regression including all factors listed in the table. Missing values handled by listwise deletion.

Supplementary Table 2

Supplementary Table 2. Chance of reaching diabetes remission depending on socioeconomic status

	Unadjusted OR (95% CI)	Adjusted OR (95%CI)[1]	Adjusted-P[1]
Education			
Primary education ≤9 years	0.92 (0.81-1.05)	0.95 (0.76-1.18)	0.634
Secondary education	Reference	Reference	Reference
Higher education ≤3 years	1.09 (0.92-1.31)	0.95 (0.72-1.27)	0.743
Higher education >3years	1.07 (0.89-1.28)	1.10 (0.81-1.48)	0.548
Disposable income			
<20 th percentile	0.93 (0.81-1.06)	0.78 (0.62-0.98)	0.033
20-50 th percentile	Reference	Reference	Reference
50-80 th percentile	1.00 (0.87-1.15)	0.94 (0.75-1.17)	0.560
>80 th percentile	0.83(0.70-0.99)	0.68 (0.51-0.90)	0.009*
Residence			
Large city or municipality	Reference	Reference	Reference
Medium-sized town or municipality	1.20 (1.06-1.35)	1.49 (1.22-1.82)	<0.001*
Small town, urban area, rural municipality	1.20 (1.05-1.37)	1.30 (1.04-1.62)	0.021
Heritage			
Swedish born, Swedish descendant	Reference	Reference	
Swedish born, non-Swedish descendant	1.07 (0.82-1.40)	0.90 (0.57-1.42)	0.655
Born outside Sweden	0.80 (0.70-0.90)	0.65 (0.53-0.80)	<0.001*

[1] adjusted for age, sex, BMI, insulin treatment at baseline, dyslipidaemia, duration of diabetes, HbA1c at baseline, and %TWL at 2 years follow-up. Missing values handled by listwise deletion.

* statistically significant after correction for multiple comparisons with the Bonferroni-Holm's method

Supplementary Table 3

Supplementary Table 3. Chance of reaching diabetes remission 2 years after surgery depending on preoperative BMI

	Total numbers	Remission			
BMI group (kg/m^2)	n (%)	n (%)	Undadjusted OR (95%CI)	Adjusted-OR (95%CI)[1]	Adj-P
<35	450	279 (62.0%)	Reference	Reference	Ref
35-40	2771	2075 (74.9%)	1.83 (1.48-2.52)	0.95 (0.67-1.34)	0.767
40-45	2706	2104 (77.8%)	2.14 (1.74-2.65)	0.90 (0.63-1.28)	0.541
45-50	1328	1087 (81.9%)	2.76 (2.18-3.50)	1.03 (0.69-1.54)	0.893
50-55	559	458 (81.9%)	2.78 (2.08-3.71)	0.65 (0.30-1.05)	0.078
55-60	166	140 (84.3%)	3.30 (2.08-5.28)	0.64 (0.33-1.37)	0.249
>60	77	68 (88.3%)	4.63 (2.25-9.52)	1.03 (0.29-3.69)	0.966

1- Multivariable logistic regression including BMI groups, age, sex, HbA1c, diabetes duration, insulin treatment, obesity-reatled disease and total weight-loss at 2 years after surgery. Missing values handled by listwise deletion.
